# *Salmonella* Outbreaks Linked to Beef, United States, 2012–2019

**DOI:** 10.1016/j.jfp.2023.100071

**Published:** 2023-03-16

**Authors:** Michelle Canning, Meseret G. Birhane, Daniel Dewey-Mattia, Hannah Lawinger, Andrea Cote, Laura Gieraltowski, Colin Schwensohn, Kaitlin A Tagg, Louise K. Francois Watkins, Misha Park Robyn, Katherine E. Marshall

**Affiliations:** 1 Centers for Disease Control and Prevention, 1600 Clifton Rd, Atlanta, GA 30333, USA; 2 Oak Ridge Institute for Science and Education, 1299 Bethel Valley Rd, Oak Ridge, TN 37830, USA; 3 Food Safety and Inspection Service, 1400 Independence Ave SW, Washington, DC 20250, USA

**Keywords:** Antimicrobial-resistance, Beef, Ground beef, Outbreaks, Prevention, *Salmonella*

## Abstract

The Centers for Disease Control and Prevention (CDC) has identified nontyphoidal *Salmonella* as one of the top five pathogens contributing to foodborne illnesses in the United States. Beef continues to be a common source of *Salmonella* outbreaks, despite the implementation of interventions at slaughter and processing facilities to reduce contamination of beef. We described *Salmonella* outbreaks linked to beef in the United States during 2012–2019, examined trends, and identified potential targets for intervention and prevention strategies. We queried CDC’s Foodborne Disease Outbreak Surveillance System (FDOSS) for all foodborne nontyphoidal *Salmonella* outbreaks linked to beef as the single contaminated ingredient or implicated food, with the date of first illness onset from 2012 to 2019. Information on antimicrobial resistance (AR) for outbreak-related isolates was obtained from CDC’s National Antimicrobial Resistance Monitoring System (NARMS). We calculated the number of outbreaks, outbreak-related illnesses, hospitalizations, and deaths overall, by beef processing category and *Salmonella* serotype. During 2012–2019, 27 *Salmonella* outbreaks were linked to beef consumption, resulting in 1103 illnesses, 254 hospitalizations, and two deaths. The most common category of beef implicated was nonintact raw, ground beef (12 outbreaks, 44%), followed by intact raw (six outbreaks, 22%). Ground beef was responsible for the most illnesses (800, 73%), both of the reported deaths, and was the source of the largest outbreak. AR data were available for 717 isolates from 25 (93%) outbreaks. Nine (36%) of these outbreaks had isolates resistant to one or more of the antibiotics tested by NARMS, of which eight (89%) contained multidrug-resistant isolates. Several outbreaks reported highlight challenges faced during investigations, areas where further research may be warranted, and opportunities to prevent future outbreaks along the farm-to-fork continuum.

CDC estimates 1.35 million nontyphoidal *Salmonella* (NTS) illnesses occur each year in the United States and identifies NTS as one of the top five pathogens contributing to foodborne illnesses in the United States (Collier et al., 2021; Scallan et al., 2011). Among the seven leading pathogens that cause foodborne illness, NTS infections resulted in the most disability-adjusted life years annually (32 900) (Scallan et al., 2015). In 2018, the U.S. Department of Agriculture’s (USDA) Economic Research Service estimated the mean total cost for illnesses, including medical costs and productivity loss, due to NTS in the United States to be $4.1 billion (U.S. Department of Agriculture Economic Research Service., 2018).

Illness caused by *Salmonella* is an ongoing concern, and it is estimated 66% of domestically acquired NTS illnesses are attributed to foodborne transmission (Beshearse et al., 2021). The yearly average number of infections during 2012–2019 did not decrease, despite objectives to reduce foodborne *Salmonella* infections as part of the Healthy People 2010 and 2020 initiatives to improve the health of all Americans (Tack et al., 2020). The incidence of foodborne *Salmonella* infections was 15 per 100 000 in 2009 (Healthy People 2010 objective: 6.8 per 100 000), and 17 per 100 000 in 2019 (Healthy People 2020 objective: 11.4 per 100 000) (National Center for Health Statistics, 2012; U.S. Department of Health and Human Services Office of Disease Prevention and Health Promotion, 2020). Not only were the objectives not met, but the incidence of infections increased.

Beef is a commonly identified source of foodborne *Salmonella* illnesses. From 2012 to 2019, beef was estimated to account for 5.7%–9.1% of all foodborne *Salmonella* illnesses (Interagency Food Safety Analytics Collaboration, 2017, 2018a, 2018b, 2018c, 2019, 2020, 2021). Further, beef was significantly more likely to be implicated in outbreaks than expected based on the frequency of beef consumption. From 2005 through 2016, while 2.3% of single-ingredient foodborne outbreaks were attributed to beef, beef accounted for 0.6% of the single-ingredient foods consumed on an average day by the United States population; this points to a potentially higher risk of contamination in beef and the need to prioritize illness and outbreak prevention (Richardson et al., 2021). Our objectives were to describe *Salmonella* outbreaks linked to beef in the United States during 2012–2019, analyze changes over time, and identify potential targets for intervention and prevention strategies.

## Materials And Methods

### Data sources and criteria.

Since 1973, CDC’s Foodborne Disease Outbreak Surveillance System (FDOSS) has collected information from state and local health departments about foodborne disease outbreaks. We queried FDOSS for all foodborne NTS outbreaks linked to beef as the single contaminated ingredient or implicated food, using a standardized categorization scheme (Richardson et al., 2017), with the date of first illness onset from 2012 to 2019. Data provided by FDOSS included the number of illnesses, hospitalizations, deaths, patient demographics, outbreak duration and geographic scope, method and setting of food preparation, traceback and recall information, and NTS serotypes for each outbreak. If multiple serotypes were reported in a single outbreak, characteristics of the outbreak were reported under each serotype in the resulting table. We searched an internal CDC database for additional information obtained during investigations of multistate outbreaks (i.e., outbreaks caused by exposures that occurred in more than one state) (U.S. Centers for Disease Control and Prevention, 2020). For a secondary analysis, we queried FDOSS for all foodborne *Salmonella* outbreak reports in which one of the multiple foods or ingredients implicated contained beef, with the date of first illness onset during 2012–2019.

We queried CDC’s National Antimicrobial Resistance Monitoring System (NARMS) for information on antibiotic susceptibility testing (AST) for outbreak-related isolates identified through FDOSS. CDC encourages health departments to submit 3-4 representative NTS clinical isolates from outbreaks for AST by the NARMS laboratory. Antibiotics tested by NARMS included amoxicillin-clavulanic acid, ampicillin, azithromycin, cefoxitin, ceftiofur (2012–2015 only), ceftriaxone, chloramphenicol, ciprofloxacin, gentamicin, kanamycin (2012–2013 only), meropenem (2016–2019 only), nalidixic acid, streptomycin, sulfisoxazole, tetracycline, and trimethoprim-sulfamethoxazole. Additionally, resistance was predicted from whole genome sequencing (WGS) data (routinely available after 2015) from select isolates sequenced and uploaded to CDC’s national surveillance system (PulseNet). Briefly, *de novo* assemblies were produced using shovill v.1.0.4 (https://github.com/tseemann/shovill) and screened for antimicrobial-resistant determinants using the ResFinder database (90% identity, 50% cutoff) (updated July 30, 2020) and the PointFinder scheme (updated August 30, 2019) for *Salmonella* spp. implemented in staramr v.0.4.0 (https://github.com/phac-nml/staramr). AST results were used to determine antimicrobial resistance (AR) when available, and AR was predicted from WGS data when an isolate did not have AST results (or for antibiotics not included on the NARMS panel) (McDermott et al., 2016). Although outbreaks can have food or environmental isolates available for screening, we are only reporting AR results from isolates cultured from human infections.

### Definitions.

An outbreak was defined by FDOSS as two or more illnesses resulting from the consumption of a common food. During 2012–2019, the primary molecular subtyping method for detecting outbreaks and defining the outbreak strain was pulsed-field gel electrophoresis (PFGE). The outbreak strain was, therefore, defined by the PFGE pattern, though for several outbreaks, WGS was used to further characterize the outbreak strain. Outbreak duration was the number of days from the date the first person became ill up to the day the last person became ill. Investigation duration was only calculated for multistate outbreaks and was calculated as the number of days from when CDC was notified of the outbreak to when CDC ended its investigation. To assess seasonality, the number of outbreaks and illnesses were aggregated by the month the first illness occurred.

For outbreaks reported during 2017–2019, food vehicles were classified as suspected or confirmed at the time they were reported to FDOSS. Briefly, vehicles are confirmed if it is a point-source outbreak linked to a meal or a single event and at least one type of evidence (e.g., epidemiologic, traceback, laboratory data) is available, or if exposures occur in multiple venues/locations and at least two types of evidence are provided (U.S. Centers for Disease Control and Prevention, 2017). For consistency, we retrospectively classified vehicles as confirmed or suspected for outbreaks that were investigated and reported before 2017 using the same methodology (U.S. Centers for Disease Control and Prevention. Date, 2017).

Beef was classified into one of the five following categories: nonintact raw, intact raw, or ready-to-eat (RTE), according to the Interagency Food Safety Analytics Collaboration (IFSAC) Food Categorization Scheme, and two other categories: other beef and unspecified beef (Richardson et al., 2017). Outbreaks for which a specific type of beef was reported, but there was no information on how it was processed, were classified as “other beef”. Outbreaks for which no specific type of beef was reported were classified as “unspecified beef”. In instances where multiple implicated beef types were reported, the least specific common category was used (Richardson et al., 2017, 2021). All nonintact raw beef was further categorized as ground beef or tenderized/injected beef.

For isolates with AST results, we defined resistance based on clinical breakpoints determined by the Clinical and Laboratory Standards Institute (CLSI) when available (Humphries et al., 2021); otherwise, NARMS breakpoints were used (U.S. Centers for Disease Control and Prevention, 2018). We defined multidrug-resistant (MDR) isolates as resistance to at least one drug from three or more CLSI antibiotic classes. Clinically significant antibiotics refers to those commonly used to treat patients with severe infections; the list of recommended treatments for *Salmonella* in humans includes ampicillin, ceftriaxone, ciprofloxacin, trimethoprim-sulfamethoxazole, and azithromycin (Committee on Infectious Diseases et al., 2021; Shane et al., 2017).

### Analyses.

We calculated the number of outbreaks, outbreak-related illnesses, hospitalizations, and deaths, by beef processing category and *Salmonella* serotype. We assessed characteristics of outbreaks including patient demographics (age and sex), geography (single-state vs. multistate), seasonality, and food preparation method and setting. We determined the number of outbreaks caused by MDR strains. Statistical tests including Fisher’s exact, chi-square, and Kruskal-Wallis were used to compare characteristics of outbreaks during the first four years (2012–2015) with the most recent four years (2016–2019). Tests were assessed at the 0.05 level of significance. SAS software, Version 9.4 (SAS Institute Inc., Cary, NC, USA) was used.

## Results

During 2012–2019, 27 *Salmonella* outbreaks were linked to beef consumption, resulting in 1103 illnesses, 254 hospitalizations, and two deaths (Table 1). A median of four outbreaks (range: 1–5), 91 illnesses (range: 9–488), and 16 hospitalizations (range: 1–132) were linked to beef each year. Information on patient age was available for 911 (83%) ill people; the largest percentage of illnesses were among those 20–49 years of age (n = 360, 40%); among the most vulnerable to serious illness, 61 (7%) were <5 years, and 61 (7%) were 75 years and older (Table 1). Of the 927 (84%) ill people with information on sex, 466 (50%) were female.

Investigators identified beef as the confirmed food vehicle in 19 (70%) outbreaks, and as a suspected vehicle in 8 (30%) outbreaks. Among the 27 outbreaks, 10 (37%) had only epidemiologic evidence, 4 (15%) had epidemiologic and laboratory evidence, 3 (11%) had epidemiologic and traceback and/or environmental investigation evidence, and 10 (37%) had epidemiologic, traceback and/or environmental investigation, and laboratory evidence implicating beef as the outbreak source.

### Multistate vs.single-state outbreaks.

**Figure 1.**
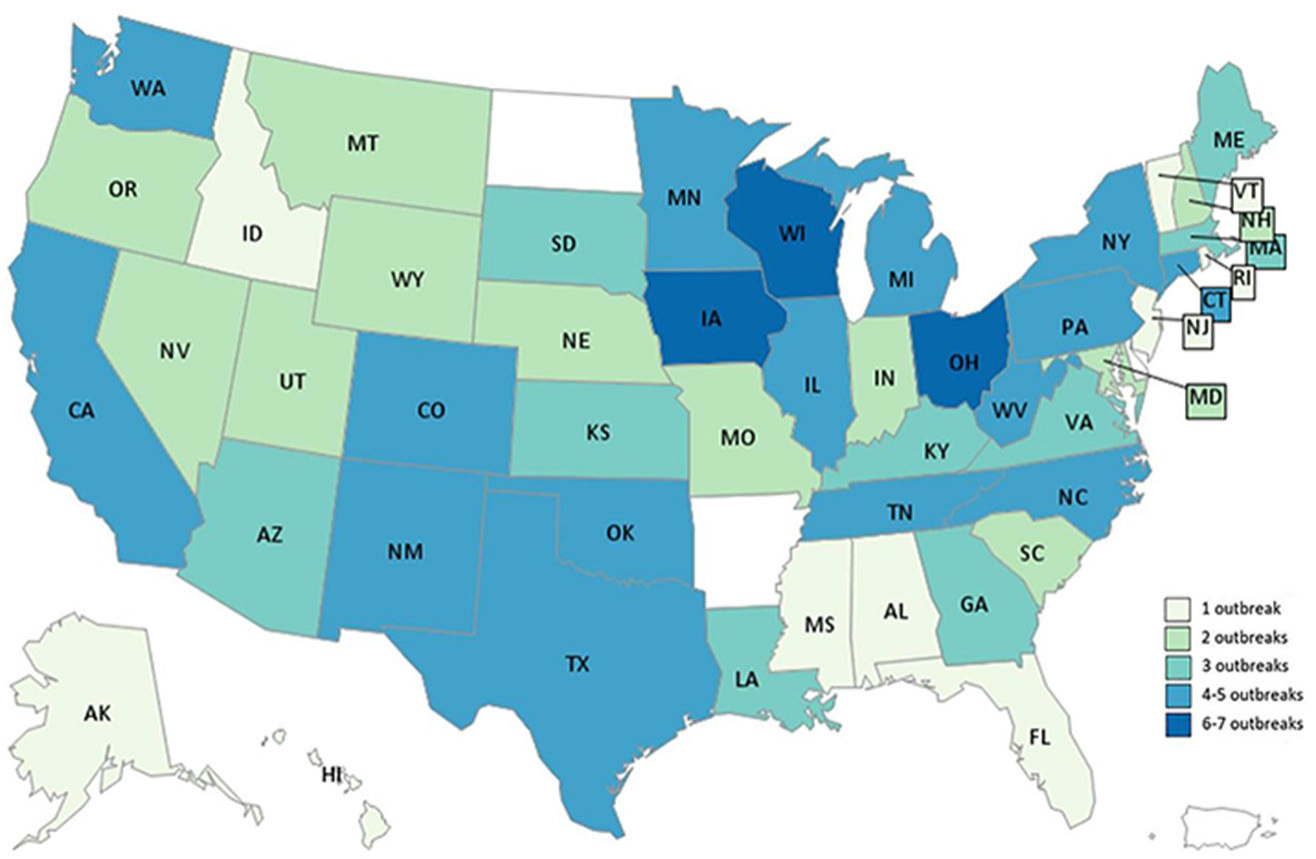
Number of *Salmonella* outbreaks linked to beef by state, United States, 2012–2019. There were 19 single-state outbreaks and 8 multistate outbreaks. Multistate outbreaks are counted as an outbreak for each state that reported a case. Single-state outbreaks occurred in 13 states: California (1), Colorado (1), Connecticut (2), Georgia (1), Minnesota (2), New Mexico (1), New York (1), Ohio (2), Oregon (1), Tennessee (2), Virginia (1), Washington (1), Wisconsin (3).

Of the 27 outbreaks, 19 (70%) were single-state outbreaks and 8 (30%) were multistate. Multistate outbreaks accounted for 763 (69%) of all illnesses. Multistate outbreaks were larger (median 48 vs. 6 illnesses), more severe (216/579 (37%) vs. 38/255 (15%) hospitalized), and longer in duration (median 110 vs. 6 days) than single-state outbreaks. Single-state outbreaks occurred in 13 states. Outbreak-related illnesses occurred in 47 states (Fig. 1).

### Beef categories.

The most common beef category implicated was nonintact raw (12 outbreaks, 44%), followed by intact raw (six outbreaks, 22%), other beef (four outbreaks, 15%), and RTE beef (two outbreaks, 7%). For three (11%) outbreaks there was not enough information to determine the unspecified beef category. All nonintact raw beef products implicated in outbreaks were ground beef. Intact raw beef included steaks (four outbreaks), ribs (one outbreak), and beef brisket (one outbreak). RTE beef included jerky and sliced roast beef deli meat. Beef in the “other” category included roast beef, ox tongue and tripe, fajita beef, and raw laab and boiled beef.

Among the specified beef types, ground beef was responsible for the most illnesses (800, 87%), both of the reported deaths, and was the source of the largest outbreak (Table 1). Outbreaks linked to intact raw beef caused the highest percentage of hospitalizations (10/26, 38%), closely followed by ground beef (221/598, 37%) (Table 1).

Information on retailer practices regarding ground beef was available for 11/12 (92%) ground beef outbreaks. Case-ready refers to meat that comes to a store packaged for sale, so the retailer does not repackage it (U.S. Centers for Disease Control and Prevention, 2017). Seven (64%) outbreaks distinguished between case-ready (three outbreaks) and not case-ready (four outbreaks). Six (55%) outbreaks had information on whether the ground beef was ground or reground by the retailer. In 3 (50%) outbreaks, ground beef was ground or reground. Additional information on whether anything was added to the beef during the grinding or regrinding was available for one outbreak; shop trim (i.e., pieces of meat remaining after cuts were removed) was added to the beef.

### Food preparation.

Information on the settings where beef was prepared was available for 26 outbreaks (96%); 24 (92%) of these had a single setting of preparation. The most common single settings of preparation were private homes (12, 46%) and restaurants (8, 31%) (Table 1). In outbreaks linked to ground beef, the most common preparation setting was a private home (eight outbreaks, 67%), whereas, in outbreaks linked to intact raw beef, restaurants were the most common preparation setting (four outbreaks, 67%). The median outbreak duration was longer for outbreaks where beef was prepared at home (75 days) compared with outbreaks where beef was prepared in a restaurant (10 days). Nine (75%) outbreaks linked to ground beef had information on food preparation; in 3 (33%) outbreaks, the ground beef was consumed raw (Supplementary Table 1).

### Changes over time.

**Figure 2.**
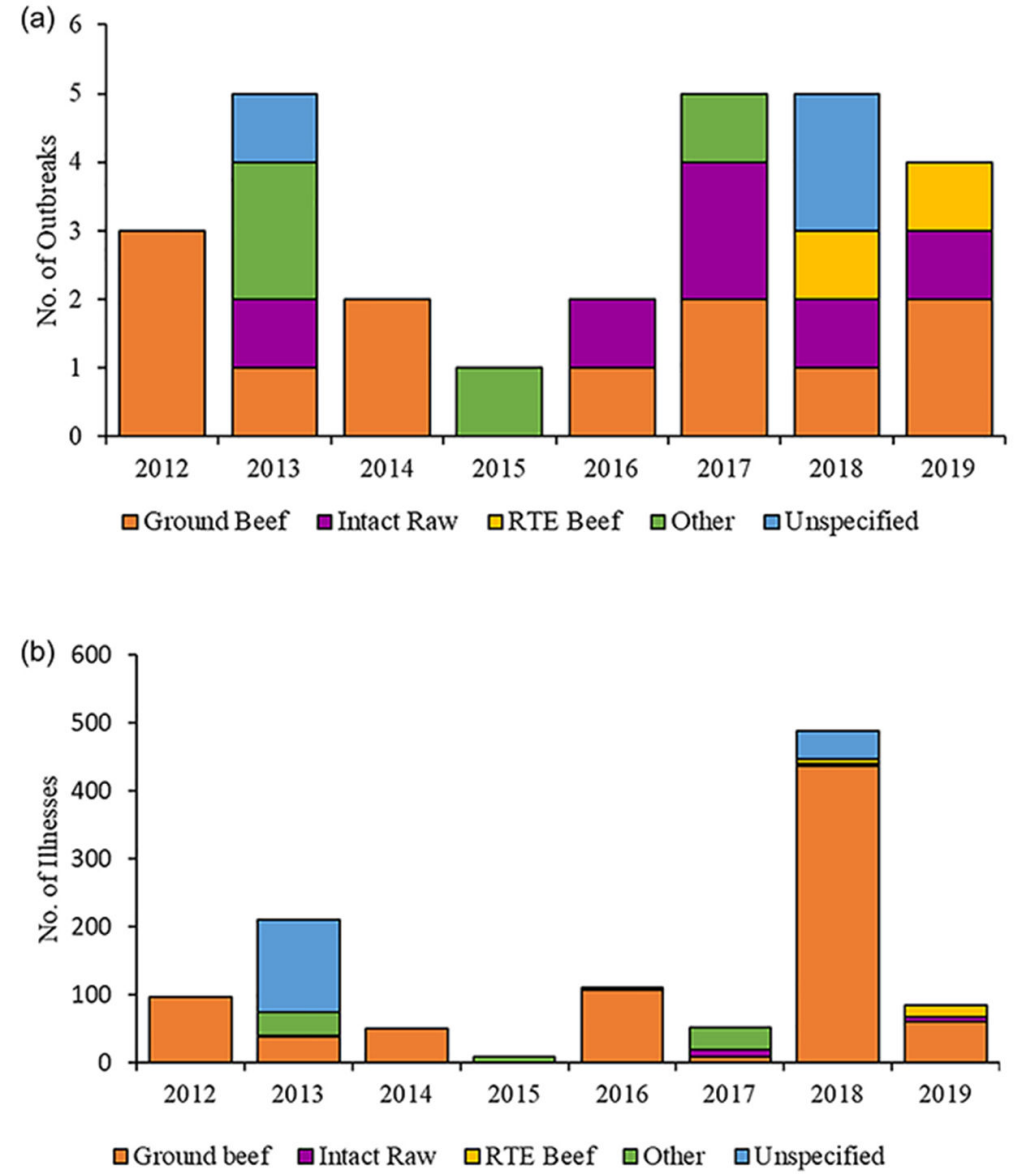
(a) Number of *Salmonella* outbreaks linked to beef, by year and beef type, United States, 2012–2019. (b) Number of *Salmonella* outbreak-related illnesses linked to beef, by year and beef type, United States, 2012–2019.

There were more outbreaks associated with beef during 2016–2019 compared to 2012–2015 (total: 16 vs. 11) (Fig. 2a), though the median yearly number of outbreaks did not differ significantly between the two time periods (4.5 vs. 2.5, p = 0.30). The median yearly number of illnesses in the two time periods was not significantly different (2016–2019 median: 98 vs. 2012–2015 median: 74, p = 0.39).

At least one outbreak linked to ground beef occurred each year, except for 2015. A median of two (range: 0–3) outbreaks and 56 (range: 0–436) illnesses were linked to ground beef each year. The median yearly number of outbreak-related illnesses linked to ground beef was higher in the second four years (45 vs. 84), although the difference was not statistically significant (p = 0.25). The largest outbreaks linked to ground beef also occurred during the second time period (Fig. 2b).

### Seasonality.

**Figure 3.**
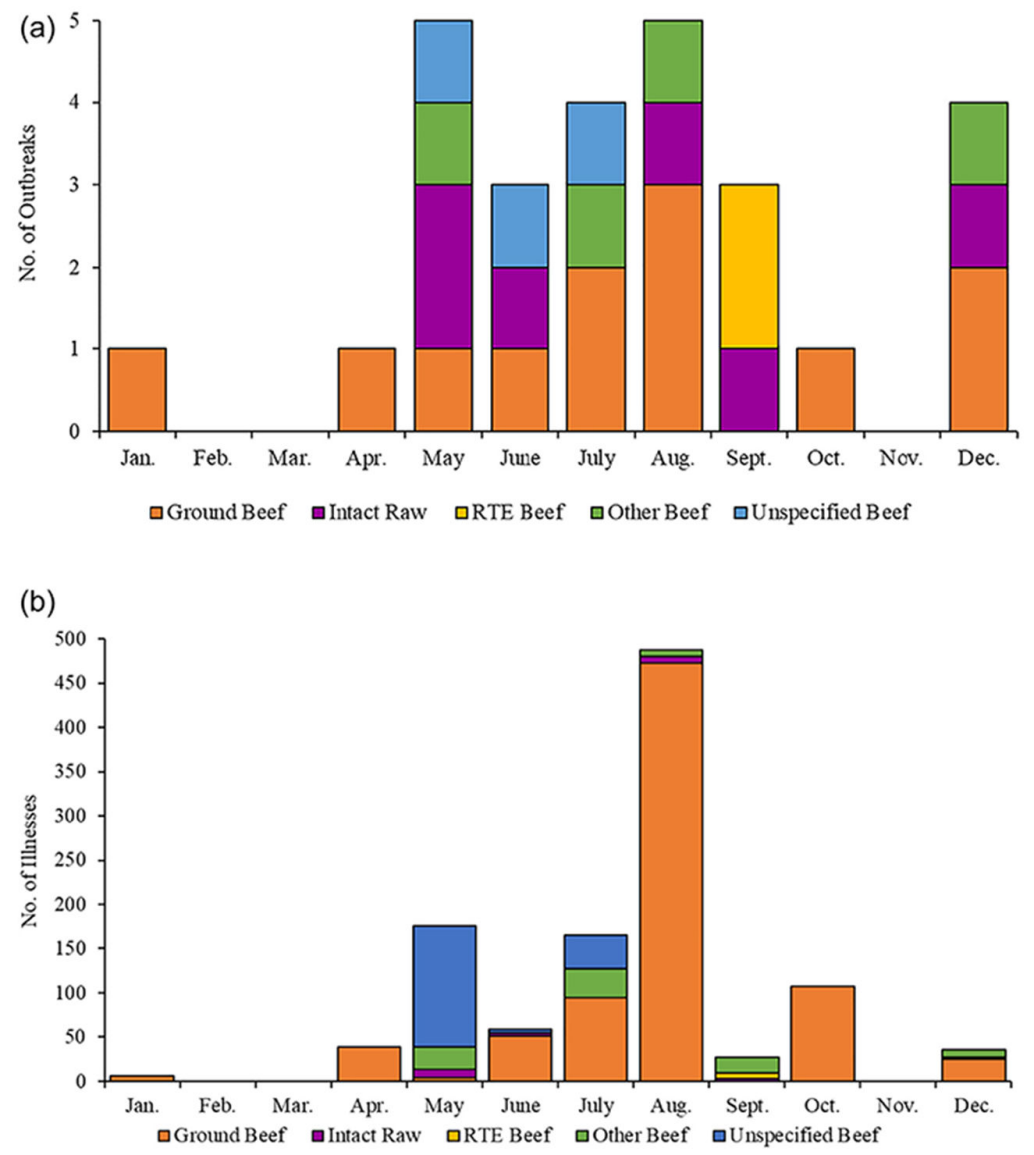
(a) Number of *Salmonella* outbreaks linked to beef, by month and beef type, United States, 2012–2019. (b) Number of *Salmonella* outbreak-associated illnesses linked to beef, by month and beef type, United States, 2012–2019.

*Salmonella* outbreaks linked to beef occurred most frequently in May and August (five outbreaks each), followed by July and December (four outbreaks each). *Salmonella* outbreaks linked to ground beef occurred in 8 of the 12 months, with the most in August (three outbreaks) (Fig. 3a). One of the two ground beef outbreaks in December was linked to a holiday tradition in Wisconsin. All cases in this outbreak consumed a traditional holiday dish that includes raw ground beef served on bread with onions, typically called a “cannibal sandwich”, at a private residence (U.S. Centers for Disease Control and Prevention, 2013). The highest reported number of outbreak-related illnesses occurred in August (488 illnesses), followed by May (176 illnesses) and July (165 illnesses) (Fig. 3b).

### Serotypes and nonhuman sources of outbreak-associated isolates.

The 27 outbreaks linked to beef were caused by 12 different *Salmonella* serotypes, with Newport (7, 26%), Typhimurium (6, 22%), and Enteritidis (3, 11%) causing more than half of the outbreaks linked to all beef types (Table 2). One outbreak was caused by two *Salmonella* serotypes: Typhimurium and Idikan. Outbreaks caused by Newport resulted in the most illnesses (732, 66%) and one of the two deaths in the reporting timeframe. Across all outbreaks, those caused by Typhimurium (33/97 (34%) hospitalized), Newport (187/572 (33%) hospitalized), Dublin (16/49 (33%) hospitalized), and Muenchen (1/3 (33%) hospitalized) were the most severe.

Among the 12 outbreaks linked to ground beef, four were caused by *Salmonella* Newport, four were Typhimurium, three were Enteritidis, and one was Dublin. The Dublin outbreak linked to ground beef was the most severe, with (9/11) 82% of patients hospitalized and one death. The largest outbreak linked to ground beef was caused by a specific strain of *Salmonella* Newport that resulted in 436 illnesses and 124 hospitalizations. This same outbreak strain previously caused an outbreak in 2016–2017 that resulted in 107 illnesses and was also linked to ground beef.

An outbreak strain was isolated from food specimens in 12 (44%) outbreaks, and from dairy cattle in one of these 12.

### Antimicrobial resistance.

**Figure 4.**
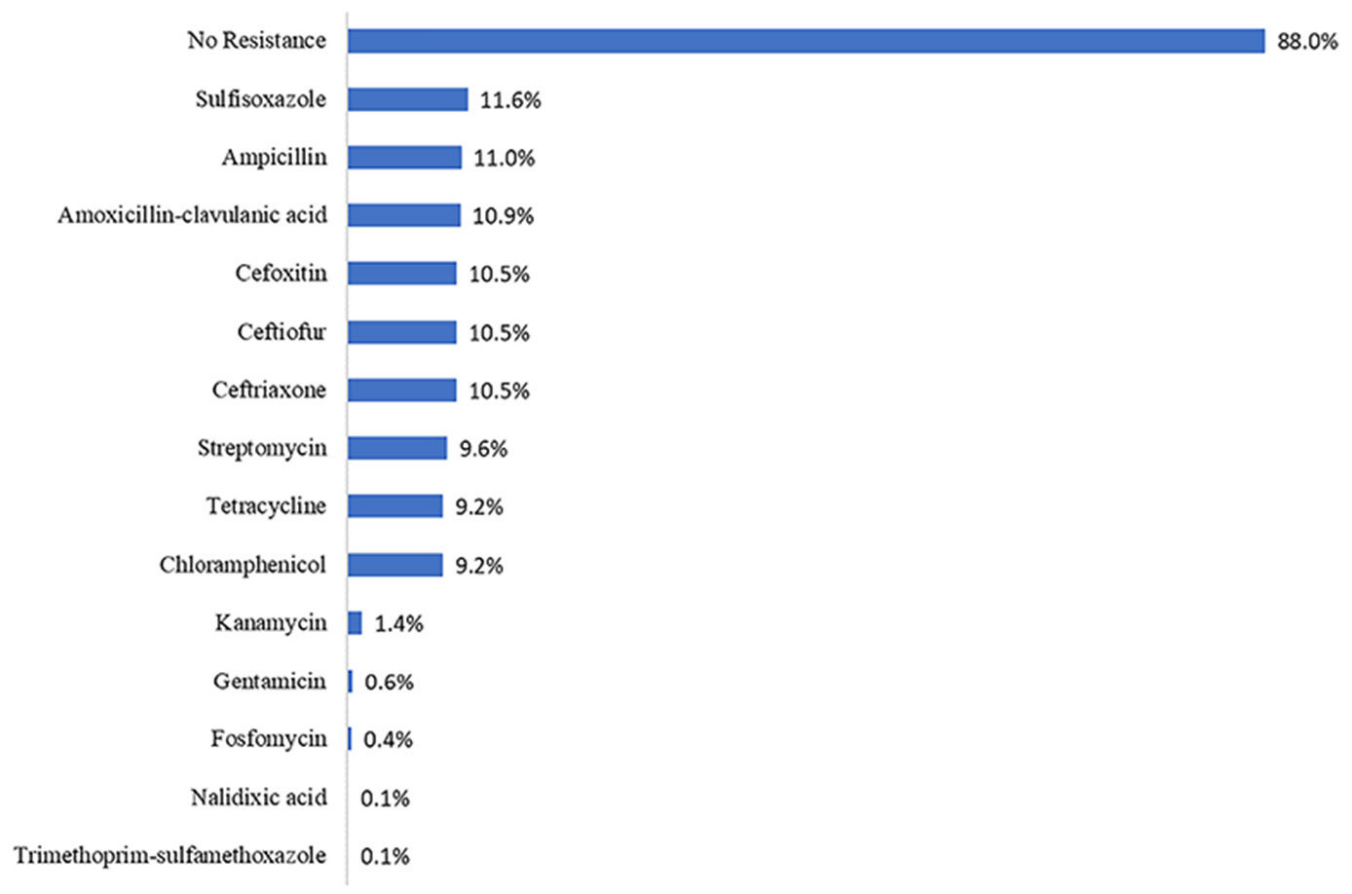
Percentage of nontyphoidal *Salmonella* clinical isolates with antimicrobial resistance^A^ from outbreaks linked to beef as the single contaminated ingredient or implicated food (n = 25), National Antimicrobial Resistance Monitoring System, (n = 117), United States, 2012–2019. ^A^ No isolates showed resistance to azithromycin, ciprofloxacin, or meropenem.

AR data were available for 717 isolates from 25/27 (93%) outbreaks. Although 88% of isolates showed no resistance, 11% showed resistance to both ampicillin and ceftriaxone, two of the antibiotics recommended for treatment (Fig. 4). Strains from 16 (64%) of the 25 outbreaks were susceptible to all antimicrobials tested (Supplemental Table 1). Nine (36%) of the 25 outbreaks contained isolates that were resistant to one or more of the antibiotics tested by NARMS, and among these nine outbreaks, eight (89%) contained MDR isolates. Among these eight, three (38%) were caused by *Salmonella* Newport, two (25%) by *Salmonella* Typhimurium, one (13%) by *Salmonella* Dublin, one (13%) by *Salmonella* Heidelberg, and one (13%) was caused by multiple serotypes. In the three MDR *Salmonella* Newport outbreaks, 92–100% of tested isolates displayed resistance to ampicillin, chloramphenicol, streptomycin, sulfamethoxazole/sulfisoxazole, tetracycline, amoxicillin-clavulanic acid, ceftriaxone, cefoxitin, and ceftiofur (Supplemental Table 1). All eight MDR outbreaks contained isolates that were resistant to at least one of the clinically significant antibiotics used in human medicine.

### Traceback and recalls.

A traceback investigation was conducted for 10 (37%; seven multistate) of the 27 outbreaks. Among these, a single, common source location was identified for four multistate outbreaks and one single-state outbreak: four slaughter/processing establishments and one retail store. For all four of these multistate outbreaks, the product was recalled, resulting in more than 12 million pounds of ground beef recalled (range: 1050 pounds–12 million pounds per outbreak). In two, the recalled ground beef was packaged as various-sized chubs; one included ground beef sold as patties, loaves, and chubs, and for another, the ground beef was packaged in clear, plastic bags.

### Outbreaks linked to multiple foods that included beef.

We identified 21 additional *Salmonella* outbreaks from 2012 to 2019 in which one of the multiple foods or ingredients implicated contained beef (Supplemental Table 1). These 21 outbreaks resulted in 542 illnesses, 97 hospitalizations, and three deaths. The median outbreak size was nine illnesses (range: 2–221). Among the 21 outbreaks, the largest occurred in 2018 (221 illnesses) and was a multistate outbreak of *Salmonella* Newport linked to U.S. beef and Latin-style soft cheese in Mexico (Plumb et al., 2019). Most infections from this outbreak were resistant to multiple antibiotics recommended for treatment, including ampicillin, azithromycin, ciprofloxacin, and trimethoprim-sulfamethoxazole; this decreased susceptibility to azithromycin had been recently detected for the first time in human isolates by NARMS surveillance in 2016 (Plumb et al., 2019). Outbreaks with vehicles containing multiple ingredients with no confirmed contaminated ingredient were smaller, more often single-state, more often in restaurants, and were shorter in duration than outbreaks linked to beef as the single contaminated ingredient or implicated food.

## Discussion

During 2012–2019, *Salmonella* outbreaks linked to beef occurred regularly and outbreak-associated illnesses were reported in almost all states. We did not observe a statistically significant increase in outbreaks or illnesses within this study period. Ground beef was the source of the most illnesses, hospitalizations, and both deaths. Further, the largest *Salmonella* outbreak linked to ground beef in the United States ever reported occurred in 2018 and resulted in the largest ground beef recall associated with an outbreak of salmonellosis. Approximately one-third of outbreaks were caused by antimicrobial-resistant strains and all but one of these were MDR. MDR strains can cause more severe outcomes in patients, particularly isolates with resistance to ampicillin, chloramphenicol, streptomycin, sulfamethoxazole/sulfisoxazole, and tetracycline, which was present in more than 50% of our MDR outbreaks (Krueger et al., 2014). We found nearly as many outbreaks in FDOSS in which one of the multiple foods or ingredients implicated was beef, as outbreaks attributed to beef as the single implicated food or contaminated ingredient. This suggests that estimates of outbreaks and illnesses attributed to beef could be underestimated, further emphasizing the need for action.

Our data suggest that focusing prevention efforts on ground beef may be especially important. Ground beef was the source of the most outbreaks, illnesses, hospitalizations, and both deaths among specified beef categories (12 outbreaks [50%], 800 illnesses [87%], 221 hospitalizations [92%]); more than half (56%) of the outbreaks caused by antimicrobial-resistant strains were linked to ground beef. Further, there were nearly as many illnesses (800 vs. 916) and more hospitalizations (221 vs. 141) linked to ground beef during 2012–2019 as there were in the 39 years prior (1973–2011, 22 outbreaks) (Laufer et al., 2015). While outbreaks were likely missed before PulseNet was launched in 1996, these data suggest that despite the implementation of interventions over the last several decades, additional improvements in ground beef safety are needed to prevent outbreaks and illnesses.

There are several reasons why ground beef may continue to contribute disproportionately to beef-associated illnesses. First, multiple carcasses contribute to the production of ground beef, allowing bacteria from one animal to be widely distributed across multiple ground beef products. Second, the grinding process allows bacteria from a contaminated surface to be blended throughout the ground beef, making it more difficult to kill internalized bacteria and making cooking to an internal temperature of 160 degrees Fahrenheit critical. Third, undercooking ground beef is common (Patil et al., 2005). Though information on how individuals linked to outbreaks prepared ground beef was not systematically assessed for this analysis, in an outbreak attributed to ground beef in 2016, 12 (36%) patients reported possibly undercooking their ground beef (Marshall et al., 2018). A third of ground beef outbreaks with information regarding preparation were linked to ground beef that was consumed raw. Lastly ground beef is a popular form of beef. In 2021, ground beef comprised 50% of beef sold at retailer meat departments by weight. (beefitswhatsfordinner.com/retail/sales-data-shooper-insights/ground-beef-sales)

At the consumer and retail level, cooking beef to an internal temperature of 160 degrees Fahrenheit, handwashing, and avoiding cross-contamination in the kitchen are important interventions to reduce levels of any *Salmonella* already present in beef and prevent illness. However, some restaurants and consumers may not know the temperature needed to thoroughly cook ground beef, may underutilize thermometers to verify the temperature, particularly for ground beef, or simply prefer consuming it undercooked. In a study that assessed restaurants in eight U.S. states, 81% of the restaurants declared a burger’s doneness using subjective measures (Bogard et al., 2013). According to the 2016 FDA Food Safety Survey, 67% of consumer respondents reported owning a thermometer; 38% reported always using it to check the temperature of roasts, and only 10% always used it to check the temperature of hamburgers (U.S. Food and Drug Administration, 2017). One study of 199 consumers reported 23% of respondents preferred their burgers pink (Phang & Bruhn, 2011). Further, the consumption of raw ground beef may be closely tied to cultural traditions; in our analysis, we identified several outbreaks linked to ground beef that was intentionally consumed raw (e.g., cannibal sandwiches and kitfo). Understanding purchasing behavior and consumers’ knowledge, awareness, perceptions, and attitudes concerning ground beef preparation and various postharvest interventions can help identify knowledge gaps and potential areas for education and is essential in aiding the development of consumer-focused messaging by public health professionals.

Ensuring the safety of beef requires multiple interventions along the entire farm-to-fork continuum, and consumer actions are only the final step. The first step in preventing contamination is appropriate sanitary dressing procedures during slaughter. Throughout slaughter and processing, implementing additional interventions that reduce possible contamination in ground beef inputs and finished ground beef may help prevent illness. Since its introduction in 1996, interventions implemented as part of the Hazard Analysis and Critical Control Point (HACCP) Systems, such as acid rinses and hot water sprays, have led to reductions in the detection of *Salmonella* and pathogenic *Escherichia coli* on cattle carcass surfaces (Dormedy et al., 2000; U.S. Department of Agriculture Food Safety and Inspection Service, 2021; Wilhelm et al., 2017; Williams et al., 2020). While these methods are effective in reducing surface bacteria, *Salmonella* can persist in the lymph nodes of cattle (Arthur et al., 2008; Gragg et al., 2013; Haneklaus et al., 2012; Webb et al., 2017) and can be incorporated into ground beef during the grinding process. Removing them during slaughter and processing may help reduce the contamination of ground beef. The United States Department of Agriculture Agricultural Marketing Service (USDA AMS) requires the removal of major lymph nodes (prefemoral, popliteal, and prescapular) for establishments to be considered as vendors for the National School Lunch Program (NSLP), in addition to implementing a zero tolerance for *Salmonella* in ground beef and requiring every lot of ground beef be tested (U.S. Department of Agriculture Agricultural Marketing Service, 2017). This combined approach appears successful in reducing contamination of ground beef for three reasons; 1) during 2006–2012, fewer NSLP samples tested positive for *Salmonella* compared with other commercial suppliers (Ollinger & Bovay, 2014); however, suppliers must prequalify to bid on NSLP contracts, potentially lowering the number of positive samples among NSLP suppliers (Ollinger & Bovay, 2014; U.S. Department of Agriculture Agricultural Marketing Service, 2017); 2) no *Salmonella* outbreaks were attributed to ground beef purchased by AMS for the NSLP during 1998–2007 (National Research Council (US) Committee, 2010); 3) we did not identify any outbreaks in school settings during 2012–2019. Additional interventions applied to finished ground beef, like irradiation, could further reduce risk (Tauxe, 2001). This added step could be particularly appealing for people who prefer to consume undercooked or raw ground beef, or for people serving ground beef to those who are at high risk of severe disease.

Prevention strategies at the preharvest stage, like bovine vaccination and biosecurity management practices, carry the potential for the greatest impact in the reduction of *Salmonella* illness, as reducing *Salmonella* at the farm level could lay the groundwork for reduction across all other levels (Edrington et al., 2020; Wilhelm et al., 2017). We identified Newport and Typhimurium as the top serotypes causing *Salmonella* outbreaks associated with beef, consistent with the previous analysis (Laufer et al., 2015). Effective vaccines that target these serotypes may help reduce the contamination of beef and prevent illness and outbreaks (Edrington et al., 2020), as demonstrated by the successful reduction of Typhimurium via vaccination within the chicken industry (Dorea et al., 2010). Further, biosecurity practices like controlling the movement of people and animals on and off farms, maintaining a closed herd, conducting microbiologic testing of animals, and implementing cleaning and disinfecting practices can help decrease the burden of *Salmonella* in these environments (Stuttgen et al., 2017).

We found that investigators were rarely able to trace implicated beef to a single processing or slaughter facility, let alone trace it back to the farm level, hindering prevention efforts. A butcher shop identified during a traceback investigation of a 2017 outbreak linked to ground beef did not keep grinding records, preventing the identification of slaughter or processing facilities that supplied the contaminated ground beef. FSIS requires all official establishments and retail stores that grind beef to maintain records regarding raw beef products (U.S. Department of Agriculture Food Safety and Inspection Service, 2015). However, even with appropriate record-keeping, beef ground at retail can come from multiple sources, further complicating the identification of a single source. Being able to trace contaminated products to a slaughter facility allows for the potential to assess controls at the facility for reducing bacteria on meat. Regulatory traceback efforts in an outbreak investigation focus on tracing back to slaughter or processing establishments. In a 2016 outbreak linked to ground beef, dairy cows were hypothesized to be the ultimate source. However, investigators were not able to trace contaminated ground beef that ill people consumed back to a source farm because multiple slaughter establishments were identified in traceback, and cows were not systematically tracked from farm to slaughter establishments, therefore, no root cause was identified (Marshall et al., 2018). The same strain caused an outbreak four times as large the following year (U.S. Centers for Disease Control and Prevention, 2019). The opportunity to trace cattle from slaughter back to source farms would better allow investigators to identify a common farm or farms and work with animal health experts to identify on-farm prevention opportunities.

Understanding the role that dairy versus beef cattle play in harboring *Salmonella* and the contamination of ground beef resulting in human illness may help identify potential public health interventions at the preharvest level. Dairy cows account for approximately 25% of U.S. nonfed beef (beef from cattle not fed feedlot rations to produce high-quality grades) (U.S. Department of Agriculture Animal and Plant Health Inspection Service, 1996). Approximately 18% of U.S. ground beef is from dairy cows (U.S. Department of Agriculture Animal and Plant Health Inspection Service, 1996). In an outbreak of *Salmonella* Newport infections in 2013, along with the consumption of ground beef, illnesses were associated with exposure to raw milk, suggesting that dairy cattle may have played an important role. The third largest outbreak in this reporting timeframe was a Newport outbreak linked to ground beef in which the outbreak strain was also identified in multiple dairy cattle from the same state; one dairy cow was sampled at a slaughter facility, and the others were sampled on a dairy farm. In our secondary analysis, the source of a 2018 Newport outbreak was beef and soft cheese, indicating dairy cattle were a likely source (Plumb et al., 2019). Some of the serotypes that were most common among outbreaks linked to beef in this study (Newport and Typhimurium) have been isolated from dairy cattle, are often MDR, and can be resistant to clinically important antibiotics (Davidson et al., 2018; Food and Drug Administration, 2022; Gragg et al., 2013). In this study, three Newport outbreaks and two Typhimurium outbreaks were MDR and were resistant to one or more clinically significant antibiotics used to treat *Salmonella* infection in humans. Identifying whether dairy or beef cattle are the underlying sources of beef contamination, particularly among outbreaks caused by antimicrobial-resistant strains, and conducting root cause investigations could inform the development of targeted interventions (The Pew Charitable Trusts, 2020).

This analysis has several limitations. First, not all outbreaks are detected or reported, and not all people who get sick with foodborne illnesses seek care, so the number of illnesses reported is likely an underestimate (Scallan et al., 2011). Second, any outbreak source, let alone a single source, is not always identified during an outbreak investigation, resulting in an underestimate of the true burden of *Salmonella* illness from contaminated beef. From our analysis of outbreaks caused by multiple foods including beef, the current estimates of attribution are likely an underestimate. However, for this secondary analysis, the number of outbreaks included could be an overestimate of the number of reported outbreaks linked to beef. Since outbreaks with vehicles containing multiple ingredients with no confirmed contaminated ingredient were included, it could be the case the contaminated ingredient was not beef but another ingredient. Lastly, we were unable to assess the impact of preharvest practices in this analysis because root cause investigations did not occur or were not reported.

Several of the *Salmonella* outbreaks linked to beef during 2012–2019 highlight challenges faced during investigations, areas where further research may be warranted, and opportunities to prevent future outbreaks along the farm-to-fork continuum. At the consumer level, characterizing who is affected by these outbreaks and their food safety behaviors helps to better inform communication and education materials. At the retail level, understanding purchasing behaviors and consumers’ knowledge about preparation, availability, and benefits of postharvest interventions, such as irradiation, can expose gaps where interventions can be applied. During slaughter and processing, further research into the role lymph node removal plays in reducing harmful bacteria is warranted. Finally, at the farm level, biosecurity and vaccination are two prevention strategies under investigation to promote herd health. A multi-layered approach is required to ensure food safety and reduce foodborne illness incidence, and steps can be taken at each level of the farm-to-fork continuum to reach the Healthy People 2030 goal of reducing infections caused by *Salmonella* commonly transmitted through food.

## Supplementary Material

Supplemental Table 1

## Figures and Tables

**Table 1:** Characteristics of *Salmonella* outbreaks associated with beef, by beef type, Foodborne Disease Outbreak Surveillance System, United States, 2012–2019

Characteristic	*N*	Ground, *n*	Intact Raw, *n*	RTE Beef, *n*	Other Types^A^ *n*	Unspecified, *n*
**Epidemiology**						
Outbreaks	27	12 (44%)	6 (22%)	2 (7%)	4 (15%)	3 (11%)
Illnesses	1103	800 (73%)	26 (2%)	24 (2%)	74 (7%)	179 (16%)
Median per outbreak (range)	13 (2–436)	32 (3–436)	4 (2–7)	12 (6–18)	17 (8–32)	38 (4–137)
Hospitalizations available^B^	834 (76%)	598 (75%)	26 (100)	23 (96%)	69 (93%)	118 (66%)
Hospitalized	254 (30%)	221 (37%)	10 (38%)	1 (4%)	9 (13%)	13 (11%)
Deaths	2	2 (100%)	0	0	0	0
Patient sex available^B^	927 (84%)	790 (99%)	26 (100%)	24 (100%)	45 (61%)	42 (23%)
Female	466 (50%)	397 (50%)	12 (46%)	22 (92%)	15 (33%)	20 (48%)
Patient age available^B^	911 (83%)	762 (95%)	26 (100%)	22 (92%)	61 (82%)	40 22%)
<1 year	17 (2%)	17 (2%)	0	0	0	0
1 to 4 years	44 (5%)	40 (5%)	0	0	2 (3%)	2 (5%)
5 to 9 years	38 (4%)	30 (4%)	0	1 (5%)	2 (3%)	5 (13%)
10 to 19 years	105 (12%)	88 (12%)	3 (12%)	1 (5%)	10 (16%)	3 (8%)
20 to 49 years	360 (40%)	278 (36%)	19 (73%)	11 (50%)	28 (46%)	24 (60%)
50 to 74 years	286 (31%)	253 (33%)	2 (8%)	7 (32%)	18 (30%)	6 (15%)
75 years and up	61 (7%)	56 (7%)	2 (8%)	2 (9%)	1 (2%)	0
**Outbreak Investigation**						
Scope						
Single state	19 (70%)	4 (33%)	6 (100%)	2 (100%)	4 (100%)	3 (100%)
Multistate	8 (30%)	8 (67%)	0	0	0	0
Duration						
Median outbreak duration (days), range	12 (1–288)	63 (2–288)	8 (1–79)	5 (3–7)	10 (2–15)	14 (6–86)
Median investigation duration^C^ (days), range	95 (60-217)	95 (60-217)				
Setting of food preparation^D^						
Private home	12	8	1			3
Restaurant	8	1	4	1	2	
Caterer	1				1	
Religious Facility	1				1	
Grocery store	1		1			
Other	1			1		
Unknown	1	1				
Multiple	2	2				

A Other beef included: roast beef, ox tongue and tripe, fajita beef, and beef, laab raw/boiled beef.

B Available refers to the number of patients the data was available for in this analysis.

C Data available for multistate investigations only.

D Setting of food preparation is not mutually exclusive.

**Table 2:** Characteristics of outbreaks caused by *Salmonella* associated with beef, by serotype, United States, 2012–2019

Characteristic	Newport *n* (%)	Typhimurium^A^ *n* (%)	Enteritidis *n* (%)	Braenderup *n* (%)	Dublin *n* (%)	Uganda *n* (%)	Heidelberg *n* (%)	Idikan^A^ *n* (%)	Infantis *n* (%)	Javiana *n* (%)	Muenchen *n* (%)	Potsdam *n* (%)	Total *N*
Outbreaks	7 (26)	6 (22)	3 (11)	2 (7)	2 (7)	2 (7)	1 (4)	1 (4)	1 (4)	1 (4)	1 (4)	1 (4)	27
Illnesses	732 (66)	129 (12)	81 (7)	11 (1)	51 (5)	43 (4)	32 (3)	6 (1)	4 (0)	8 (1)	3 (0)	9 (1)	1103
Hospitalizations available	572	97	12	11	49	42	28	6	4	7	3	9	834
Hospitalizations (% of available by serotype)	187 (33)	33 (34)	3 (25)	3 (27)	16 (33)	3 (7)	6 (21)	0 (0)	1 (25)	0 (0)	1 (33)	1 (11)	
Hospitalizations (% of total)	187 (74)	33 (13)	3 (1)	3 (1)	16 (6)	3 (1)	6 (2)	0 (0)	1 (0)	0 (0)	1 (0)	1 (0)	254
Deaths	1 (50)	0 (0)	0 (0)	0 (0)	1 (50)	0 (0)	0 (0)	0 (0)	0 (0)	0 (0)	0 (0)	0 (0)	2
Beef Type													
Ground	4 (33)	4 (33)	3 (25)	0 (0)	1 (8)	0 (0)	0 (0)	0 (0)	0 (0)	0 (0)	0 (0)	0 (0)	12
Intact Raw	1 (17)	1 (17)	0 (0)	2 (33)	0 (0)	0 (0)	0 (0)	0 (0)	1 (17)	0 (0)	1 (17)	0 (0)	6
RTE Beef	0 (0)	1 (50)	0 (0)	0 (0)	0 (0)	1 (50)	0 (0)	1 (50)	0 (0)	0 (0)	0 (0)	0 (0)	2
Other^B^	0 (0)	0 (0)	0 (0)	0 (0)	0 (0)	1 (25)	1 (25)	0 (0)	0 (0)	1 (25)	0 (0)	1 (25)	4
Unspecified	2 (67)	0 (0)	0 (0)	0 (0)	1 (33)	0 (0)	0 (0)	0 (0)	0 (0)	0 (0)	0 (0)	0 (0)	3
Antibiotic Resistance Available	7 (100)	5 (83)	2 (67)	2 (100)	2 (100)	2 (100)	1 (100)	1 (100)	1 (100)	1 (100)	1 (100)	1 (100)	25
Any Resistance (% of available)	4 (57)	3 (60)	0 (0)	0 (0)	1 (50)	0 (0)	1 (100)	1 (100)	0 (0)	0 (0)	0 (0)	0 (0)	9
MDR (% of available)	3 (43)	3 (60)	0 (0)	0 (0)	1 (50)	0 (0)	1 (100)	1 (100)	0 (0)	0 (0)	0 (0)	0 (0)	8

A One outbreak in RTE Beef was serotyped as Typhimurium and Idikan. This outbreak is classified in the table under each of the serotypes. This outbreak had 6 illnesses, 0 hospitalizations, and 0 deaths.

B Other beef included: roast beef, ox tongue and tripe, fajita beef, and beef, laab raw/boiled beef.
